# Complete Genome Sequence of Bacillus amyloliquefaciens EA19, an Endophytic Bacterium with Biocontrol Potential Isolated from Erigeron annuus

**DOI:** 10.1128/MRA.00753-21

**Published:** 2021-09-30

**Authors:** Fan-song Zeng, Bin Yuan, Wen-qi Shi, Shuang-jun Gong, Li-bo Xiang, Da-zhao Yu, Li-jun Yang

**Affiliations:** a Hubei Key Laboratory of Crop Disease, Insect Pests and Weeds Control, Wuhan, Hubei Province, People’s Republic of China; b Key Laboratory of Integrated Pest Management on Crops in Central China, Ministry of Agriculture, Wuhan, Hubei Province, People’s Republic of China; c Institute of Plant Protection and Soil Fertilizer, Hubei Academy of Agricultural Sciences, Wuhan, Hubei Province, People’s Republic of China; University of Arizona

## Abstract

Bacillus amyloliquefaciens strain EA19 is an endophyte isolated from Erigeron annuus with antifungal activity against Blumeria graminis f. sp. *tritici*, Magnaporthe oryzae, and Fusarium graminearum. The genome sequence of this strain is 3.96 Mb and contains 3,421 coding sequences, which will facilitate an understanding of the mechanisms of biocontrol.

## ANNOUNCEMENT

Endophytic bacteria play an important role in suppressing plant diseases (1). Bacillus amyloliquefaciens, a plant-associated bacterium, can be found in plant organs and exhibits antifungal activities against plant pathogens (2–4). Genome analysis helps to elucidate the gene clusters involved in the biosynthesis of active secondary metabolites of B. amyloliquefaciens (4).

**FIG 1:**
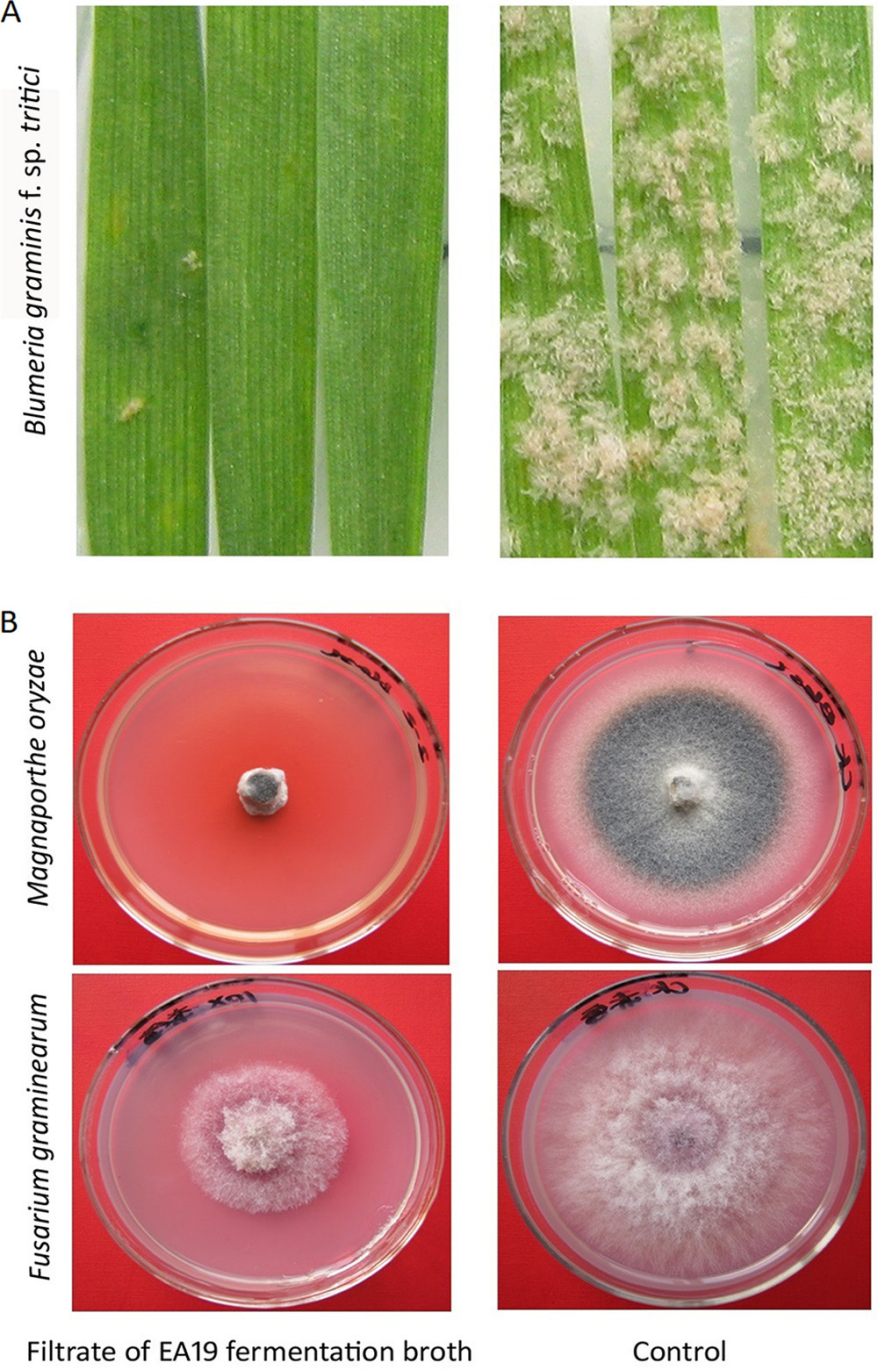
Antifungal capacities of EA19. (A) Inhibitory activities of filtrate of EA19 fermentation broth against the wheat powdery mildew pathogen Blumeria graminis f. sp. *tritici*, 10 days postinoculation on Chancellor, a susceptible wheat line. The suspension of EA19 fermentation broth was filtrated through 0.22-μm sterile syringe filters (Durapore PVDF membrane; Millipore). The culture filtrate was sprayed onto the wheat leaf segments before B. graminis f. sp. *tritici* conidia inoculation. (B) Antifungal test of filtrate of EA19 fermentation broth against the rice blast pathogen Magnaporthe oryzae and the Fusarium head blight pathogen Fusarium graminearum. The two pathogens were cultured on potato dextrose agar medium blended with 10% (vol/vol) filtrate. The control indicates the treatment with LB liquid.

Strain EA19 was isolated from the healthy stems of annual fleabane (Erigeron annuus) plants collected at the flowering stage in 2010 through the surface sterilization method (5). A single isolated colony was cultured on Luria-Bertani (LB) solid medium at 30°C for 24 h. Bacterial identification was performed using 16S rRNA and *gyrA* gene sequencing (6). Phylogenetic analysis of the two genes identified EA19 as a B. amyloliquefaciens subsp. strain (Fig. S1; https://figshare.com/search?q=10.6084%2Fm9.figshare.15145227). The culture supernatant from a single colony was incubated in LB liquid at 30°C and 180 rpm for 48 h. The fermentation broth was centrifuged at 10,000 × *g* for 5 min and then filtered through 0.22-μm sterile syringe filters (Durapore polyvinylidene fluoride [PVDF] membrane; Millipore). Antagonism tests against Blumeria graminis f. sp. *tritici* isolate W14 *in planta* and against Magnaporthe oryzae Guy11 and Fusarium graminearum PH-1 in plates were performed using the cell-free culture filtrate with detached leaf method and agar dilution method, respectively (7–9). These fungi are among the top 10 fungal pathogens in plant pathology and cause wheat powdery mildew, rice blast, and Fusarium head blight, respectively (10). The filtrate has inhibitory effects on the growth of the three pathogens (Fig. 1). Here, we report the complete genome sequence of B. amyloliquefaciens strain EA19 and its annotation.

The pellet from the fermentation broth above was harvested and genomic DNA was extracted using a QIAamp DNA extraction kit (Qiagen). The DNA library was prepared using a rapid Oxford Nanopore Technologies (ONT) sequencing kit (SQK-RAD004) and then sequenced using FLO-MIN106 flow cells on a GridION instrument (ONT, UK). Adaptor trimming and data filtering were conducted using Porechop v0.2.3 and Filtlong v0.2.0 software. Meanwhile, an Illumina library was constructed using the TruSeq Nano DNA low-throughput (LT) library prep kit (Illumina) and yielded high-quality short paired-end reads (150 bp) on the NovaSeq 6000 sequencing platform. The ONT library contained 588,025 reads with a total length of 1,884,340,507 bp, a subread *N*_50_ value of 5,804 bp, and an average coverage of 475×. Sequencing of the Illumina library generated 3,382,798 reads with a total length of 1,014,839,400 bp and an average coverage of 256×. *De novo* assembly of the genome sequence was performed using the Hierarchical Genome Assembly Process (HGAP4) workflow (11), followed by polishing with the Illumina reads. The assembled genome sequence was circularized using Minimus2 (12). NCBI PGAP v5.2 software was used for the genome annotation (13). Default parameters were used for all software unless otherwise specified.

The EA19 genome comprises a circular chromosome and one circular plasmid (pEA19). The 3,964,177-bp circular chromosome (G+C content, 46.5%) consists of 3,421 protein-coding genes, 368 pseudogenes, 27 rRNA genes, 86 tRNA genes, and 8 gene islands. The circular plasmid consists of 16,843 bp (G+C content, 40.2%) and harbors 9 protein-coding genes without pseudogenes.

### Data availability.

The genome sequence has been deposited in GenBank under the accession number CP079834. The GenBank accession number for the plasmid is CP079835. The sequence data can be found in the Sequence Read Archive under the accession numbers SRX11361551 and SRX11361552.
